# Percutaneous closure versus medical therapy for stroke with patent foramen Ovale: a systematic review and meta-analysis

**DOI:** 10.1186/s12872-018-0780-x

**Published:** 2018-03-02

**Authors:** Xin-Lin Zhang, Li-Na Kang, Lian Wang, Biao Xu

**Affiliations:** Department of Cardiology, Affiliated Drum Tower Hospital, Nanjing University School of Medicine, 321 Zhongshan Road, Nanjing, 210008 China

**Keywords:** Percutaneous closure, Medical therapy, Cryptogenic stroke, Patent foramen ovale, Meta-analysis

## Abstract

**Background:**

Patent foramen ovale (PFO) closure has emerged as a secondary prevention option in patients with PFO and cryptogenic stroke. However, the comparative efficacy and safety of percutaneous closure and medical therapy in patients with cryptogenic stroke and PFO remain unclear.

**Methods:**

Randomized controlled trials (RCTs) and comparative observational studies that compared PFO closure against medical therapy, each with a minimal of 20 patients in the closure arm and 1-year follow-up were included.

**Results:**

We analyzed 6961 patients from 20 studies (5 RCTs and 15 observational studies) with a median follow-up of 3.1 years. Moderate-quality evidence showed that PFO closure was associated with a significantly lower incidence of the composite outcome of ischemic stroke, transient ischemic attack (TIA), or all-cause death (odds ratio [OR]: 0.57; 95% confidence interval [CI]: 0.38 to 0.85; *P* = 0.006), mainly driven by lower incidence of stroke (OR: 0.39; 95% CI: 0.24 to 0.63; *P* < 0.001). The numbers needed to treat were 43 and 39 for the composite outcome and recurrent ischemic stroke respectively. PFO closure increased the risks for atrial fibrillation or atrial flutter (OR: 5.74; 95% CI: 3.08 to 10.70; *P* < 0.001; high-quality evidence) and pulmonary embolism (OR: 3.03; 95% CI: 1.06 to 8.63; *P* = 0.038; moderate-quality evidence), with the numbers needed to harm being 30 and 143 respectively. The risks for TIA, all-cause death, and major bleeding were not statistically different. Analyses limited to RCTs showed similar findings, as did a series of other subgroup analyses.

**Conclusion:**

In conclusion, PFO closure reduced the incidences of stroke and the composite outcome of ischemic stroke, TIA, or all-cause death, but increased risks for atrial fibrillation or atrial flutter and pulmonary embolism compared with medical therapy.

**Electronic supplementary material:**

The online version of this article (10.1186/s12872-018-0780-x) contains supplementary material, which is available to authorized users.

## Background

Among the 800,000 ischemic strokes that occur in the United States each year, up to 30 to 40% have no undetermined cause and are termed as cryptogenic [1]. Patent foramen ovale (PFO) was presented in 15 to 25% of the general adult population, but the prevalence was 2 to 3 times higher in patients with cryptogenic stroke [2, 3]. Paradoxical embolism via a PFO is one the potential causes of cryptogenic stroke in these patients [1, 4]. PFO closure has, therefore, emerged as a secondary prevention option in patients with PFO and cryptogenic stroke.

Three previously published randomized controlled trials (RCTs)—CLOSURE I (Evaluation of the STARFlex Septal Closure System in Patients with a Stroke and/or Transient Ischemic Attack due to Presumed Paradoxical Embolism) [5], PC (Percutaneous Closure of Patent Foramen Ovale in Cryptogenic Embolism) [6], and RESPECT (Randomized Evaluation of Recurrent Stroke Comparing PFO Closure to Established Current Standard of Care Treatment) trials [7]—all failed to show superiority of closure over medical therapy. They were insufficient to draw any conclusion because sample size of the study cohorts and event rates were lower than anticipated. A number of meta-analyses, almost all based on only these 3 RCTs, have yielded different conclusions, with some showing possible borderline benefits of closure and others showing no benefit depending on how to carry out the analysis [8–11]. The clinical benefits of PFO closure relative to medical therapy remain inconclusive. With new data recently reported from 2 additional RCTs and the extended follow-up data of the RESPECT trial, we performed an updated meta-analysis to offer a clearer picture of the efficacy and safety of PFO closure compared with medical therapy. We also included and provided separate analysis of comparative observational studies to complement findings from RCTs.

## Methods

We reported the meta-analysis in accordance with the Preferred Reporting Items for Systematic Reviews and Meta-Analyses (PRISMA) statement (Additional file 1) [12].

### Data sources and searches

We searched several electronic databases, including MEDLINE via PubMed, EMBASE, and the Cochrane Central Register of Controlled Trials from their inception to September 15, 2017, without imposing any language restriction. The following keywords and search terms were used: patent foramen ovale, PFO, stroke, closure, and occlude. We also manually checked reference lists of retrieved primary studies, relevant reviews and meta-analyses.

### Study selection

Two reviewers (X.Z. and L.K.) independently screened titles and abstracts of identified studies. Full-text of each potentially relevant study was obtained for further assessment for inclusion. Discrepancies were resolved by consensus. To be included, studies had to be randomized controlled trials (RCTs) or comparative observational studies making head-to-head comparison of patent foramen ovale closure with medical therapy in patients with cryptogenic stroke. All studies had to report at least one outcome of interest, have a minimal of 20 patients in the device closure arm and 1-year follow-up.

### Outcome measures

The primary outcome was the composite outcome of ischemic stroke, transient ischemic attack (TIA), or all-cause death; some studies also included peripheral embolism in this composite outcome. Secondary endpoints included recurrent ischemic stroke, TIA, all-cause death, atrial fibrillation (AF) or atrial flutter, pulmonary embolism, major bleeding and any serious adverse events.

### Data extraction and quality assessment

Two investigators independently extracted data from each study, which included the following items: study name, number of patients, follow-up duration, patient demographic and clinical data and outcome events. The same reviewers independently assessed the quality of each randomized trial according to the Cochrane Collaboration guideline [13] and each observational study with the Newcastle-Ottawa Scale [14]. Discrepancies were resolved through discussion and consensus.

### Grading of evidence

Two reviewers graded the overall methodological quality of each pooled analysis using the Grading of Recommendations, Assessment, Development and Evaluation (GRADE) approach, which takes into account issues related to risk of bias, inconsistency, indirectness, imprecision, and publication bias. The quality of evidence was judged as high, moderate, low or very low, using GRADEpro version 3.6 (GRADEpro GDT).

### Data synthesis and statistical analysis

We performed intention-to-treat analysis whenever possible. The longest follow-up data from individual trials were used. Odds ratios (OR) and their corresponding confidence intervals were calculated for each study and pooled with random- (DerSimonian-Laird method) or fixed- (Mantel-Haenszel method) effect models according to heterogeneity detected across studies [15]. Heterogeneity was analyzed by means of the *I*^*2*^ statistic and the χ2-based Q test [16]. The cut points were *I*^*2*^ > 50% or P of the χ2 test < 0.1. In case there was no outcome event in one of the treatment arms, the treatment arm continuity correction was applied [17]. Publication bias was assessed by visually inspecting the funnel plots and by performing Begg’s and Egger’s tests. To explore the robustness of our findings, we conducted a series of subgroup analyses based on the study design (RCT or observational study), number of patients (≥ 400 patients or < 400 patients) and duration of follow-up (≥ 3 years or < 3 years). The number needed to treat or number needed to harm was calculated from randomized trials for risk estimates where risk difference was significant, with the method from meta-analytical estimates but not treating the data as if it all arose from a single trial because the latter is susceptible to Simpson’s paradox [18, 19]. In randomized trials, several prespecified subgroup analyses were reported, which included gender (male or female), age (< 45 years or ≥ 45 years), entry event (stroke or TIA), shunt size (large or small to moderate), and atrial septal aneurysm (present or absent at enrollment). We directly extracted and performed pooled analyses of these data. We also conducted meta-regression analysis to estimate the effects of covariates, including percent of moderate to severe PFO, atrial septal aneurysm, index event of stroke, and anticoagulation in medical treatment, on major outcomes of interests. For the effect estimate, a 2-tailed *P* value less than 0.05 was considered statistically significant. Data were analyzed with Stata 12.0 (StataCorp).

## Results

### Study selection and characteristics

We identified 1408 citations through database searching. After complete evaluation, 20 studies published in 21 articles were included in the final analysis (Additional file 2: Figure S1) [5–7, 20–37]. Five studies were RCTs [5, 6, 20–22] and 15 were comparative observational studies [23–37]. A total of 6961 patients receiving device closure (*n* = 3375) or medical therapy (*n* = 3586) were included in the analysis. Sample size ranged from 92 to 980, the mean age from 35.3 to 54.0 years, the proportion of male patients from 42.0% to 76.8%, the median duration of follow-up from 1.2 to 9.0 years. The percent of index event of stroke ranged from 30% to 100%, percent of atrial septal aneurysm from 7.1% to 51.7%, percent of moderate to severe PFO from 10.5% to 100%. Three studies [21–23] exclusively used antiplatelet therapy in the medical therapy group while others used antiplatelet or anticoagulation therapy or a combination of these two antithrombotic treatments. The percent of patients receiving anticoagulation therapy was mainly reported in randomized trials, ranging from 0 to 34.2%. In most cases, closure patients received a mixed type of occlude devices, except those in 5 studies [6, 20, 25–27] that exclusively used Amplatzer PFO Occluder and those in 1 study [5] that exclusively used STARFlex Septal Closure System. Main baseline characteristics for each study are presented in Table 1. Other study characteristics are presented in Additional file 2: Table S1 to S3. The definitions of the composite outcome, major bleeding, recurrent stroke and TIA are presented in Additional file 2: Table S4.

**Table 1:** Baseline characteristics of included studies

Study	Year	No. of patients (Device/medicine)	Follow-up, ys	Device closure	Medical therapy	Age, ys	Male, %	Diabetes, %	Hypertension, %	Dyslipidaemia, %	Current Smoker, %	Moderate to severe PFO, %	Atrial septal aneurysm, %	Index event of stroke, %
CLOSURE I	2012	909 (447/462)	2	PFO closure (STARFlex)	Antiplatelet therapy, warfarin, or both	45.5	51.8	7.8	31	44.1	15.2	52.9	36.6	72
PC Trial	2013	414 (204/210)	4.1	PFO closure (Amplatzer)	Antiplatelet therapy or anticoagulation	44.5	49.8	2.7	25.8	27.1	23.9	65.6	23.7	79.2
RESPECT	2017	980 (499/481)	5.9	PFO closure (Amplatzer)	Antiplatelet therapy or warfarin	45.4	54.7	7.5	31.4	39.5	13.3	75.2	35.6	100
CLOSE	2017	473 (238/235)	5.3	PFO closure (Amplatzer, Intrasept, Premere, Starflex, etc.) plus antiplatelet therapy	Antiplatelet therapy	43.4	59	2.5	10.7	13.9	29	100	32.8	100
Gore REDUCE	2017	664 (441/223)	3.2	PFO closure (Helex, Cardioform) plus antiplatelet therapy	Antiplatelet therapy	45.1	60.1	4.2	25.6	NA	13.3	81.3	20.4	100
Wahl, et al	2012 [23]	206 (103/103)	9	Amplatzer, STAR, Sideris Buttoned, etc	Antiplatelet therapy	49.2	53.8	3.9	26.2	32.1	30.6	93.7	23.8	68.9
Alushi, et al	2014 [24]	418 (262/156)	5.9	Amplatzer, Cardia Star	Anticoagulant and/or antiplatelet therapy	48.5	52.1	5	43.8	50	26.3	51.4	33.5	57.7
Pezzini, et al	2016 [25]	521 (206/315)	3	Amplazer	Antiplatelet therapy or warfarin	35.3	47.6	1	13.8	20.3	32.5	20	NA	100
Casaubon, et al	2007 [28]	108 (47/61)	2.7	CardioSEAL and Amplatzer ASD Occluder	Antiplatelet therapy or warfarin	46	53	6	17	23	15	NA	24	69
Faggiano, et al	2012 [29]	446 (99/347)	4.5	NA	Antiplatelet therapy or warfarin	50	42	NA	NA	NA	NA	10.5	26.2	30.5
Horner, et al	2013 [31]	188 (97/79)	2	Amplatzer, CardiaStar, etc. plus antiplatelet therapy	Antiplatelet therapy or anticoagulation	42.4	51.6	3.4	25	31.3	36.9	NA	12.5	57.4
Kim, et al	2017 [32]	158 (67/91)	1.2	Amplatzer, GORE Septal Occluder	Antiplatelet therapy or anticoagulation	47.7	71.4	10.1	28.6	23.8	45.8	NA	7.1	86.3
Mazzucco, et al	2012 [34]	102 (50/52)	2.1	Amplatzer PFO Occluder, Amplatzer Cribriform occluder, BioSTAR	NA	42.6	58.8	2	17.7	33.3	20.6	NA	NA	74.5
Lee, et al	2010 [33]	184 (22/159)	3.5	Amplatzer, CardioSeal	Antiplatelet therapy or anticoagulation	41	72.9	14.9	46.4	24.9	36.5	18.2	10.5	100
Moon, et al	2016 [26]	164 (72/92)	1.8	Amplatzer	Antiplatelet therapy or anticoagulation	45.3	76.8	11.6	34.1	11	58.5	71.3	NA	94.5
Mirzada, et al	2015 [35]	314 (151/163)	5	AMPLATZER, BioSTAR, Solysafe, etc	Antiplatelet therapy or anticoagulation	54	62	6	29	22	15	NA	49	68
Schuchlenz, et al	2005 [37]	280 (167/113)	2.7	Amplatzer, CardioSEAL, STARflex	Antiplatelet therapy or anticoagulation	44	53.6	7.1	18.6	17.1	11	65.7	24.6	37.8
Paciaroni, et al	2011 [36]	238 (121/117)	2	Amplatzer, PFOStar, CardioSEAL, etc	Antiplatelet therapy or anticoagulation	42.2	49.6	2.1	19.7	19.3	29.8	NA	51.7	68.9
Thanopoulos, et al	2006 [27]	92 (48/44)	2	Amplatzer	Antiplatelet therapy	43	52.2	3.2	19.6	27.2	18.5	NA	25	67.4
Harrer, et al	2006 [30]	117 (34/83)	4.3	Amplatzer, CardioSEAL and PFOSta	Antiplatelet therapy or anticoagulation	51.1	57.3	4	23.4	8.1	21	19.4	24.2	67.7

In two studies [20, 22], the method of random sequence generation was not reported, so they were judged as being of unclear risk of bias. Blinding of personnel and participants was not possible for all trials and thus was judged as high risk of bias. All trials had blinded outcome adjudication; and the risk for detection bias, attrition bias, reporting bias and other bias were generally low. All comparative observational studies scored well on patient selection and outcome, but only 3 controlled for important confounding factors [23–25]. Detailed quality assessment of the included studies is summarized in Additional file 2: Table S5 and S6).

### Composite outcome ischemic stroke, TIA, or all-cause death

**Fig. 1:**
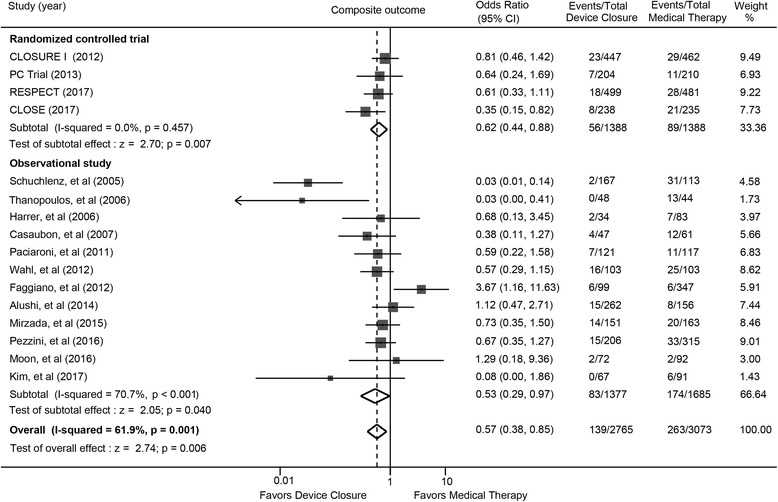
The composite outcome of recurrent stroke, TIA and all-cause death with device closure versus medical therapy. CLOSURE I = Evaluation of the STARFlex Septal Closure System in Patients with a Stroke and/or Transient Ischemic Attack due to Presumed Paradoxical Embolism; CLOSE = the Patent Foramen Ovale Closure or Anticoagulants versus Antiplatelet Therapy to Prevent Stroke Recurrence; PC = Percutaneous Closure of Patent Foramen Ovale in Cryptogenic Embolism; RESPECT = Randomized Evaluation of Recurrent Stroke Comparing PFO Closure to Established Current Standard of Care Treatment

Percutaneous closure was associated with significantly lower risk for the composite outcome of ischemic stroke, TIA, or death from any cause (OR: 0.57; 95% CI: 0.38 to 0.85; *P* = 0.006) compared with medical therapy (Fig. 1). Limiting the analyses to RCTs, the results were similar (OR: 0.62; 95% CI: 0.44 to 0.88; *P* = 0.007); the number needed to treat was 43. A statistically significant lower risk was also observed in observational studies (OR: 0.53; 95% CI: 0.29 to 0.97; *P* = 0.040), showing no significant difference with randomized trials (*P* value for interaction 0.90). Low and substantial heterogeneity was detected in randomized trials and observational studies respectively. Pooled analysis from 5 randomized trials and 3 adjusted observational studies showed very similar finding (OR: 0.64; 95% CI: 0.49 to 0.83; *P* = 0.001) (Additional file 2: Figure S2).

### Recurrent ischemic stroke

**Fig. 2:**
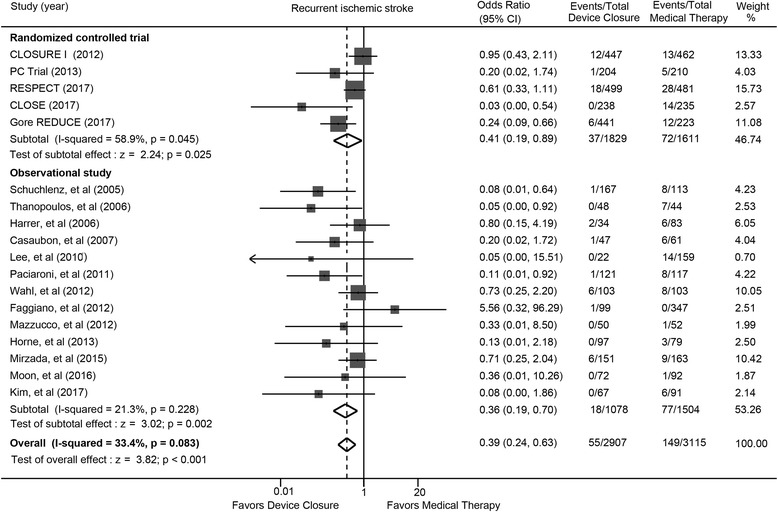
Recurrent ischemic stroke with device closure versus medical therapy. Gore CLOSURE I = Evaluation of the STARFlex Septal Closure System in Patients with a Stroke and/or Transient Ischemic Attack due to Presumed Paradoxical Embolism; CLOSE = the Patent Foramen Ovale Closure or Anticoagulants versus Antiplatelet Therapy to Prevent Stroke Recurrence; PC = Percutaneous Closure of Patent Foramen Ovale in Cryptogenic Embolism; REDUCE = Gore HELEX Septal Occluder and Antiplatelet Medical Management for Reduction of Recurrent Stroke or Imaging-Confirmed TIA in Patients with Patent Foramen Ovale; RESPECT = Randomized Evaluation of Recurrent Stroke Comparing PFO Closure to Established Current Standard of Care Treatment

Percutaneous closure significantly reduced the risk for recurrent ischemic stroke compared with medical therapy (OR: 0.39; 95% CI: 0.24 to 0.63; *P* < 0.001) (Fig. 2). Similar finding were found in randomized trials (OR: 0.41; 95% CI: 0.19 to 0.89; *P* = 0.025) and observational studies (OR: 0.36; 95% CI: 0.19 to 0.70; *P* = 0.002). The number needed to treat derived from randomized trials was 39.

### Transient ischemic attack

**Fig. 3:**
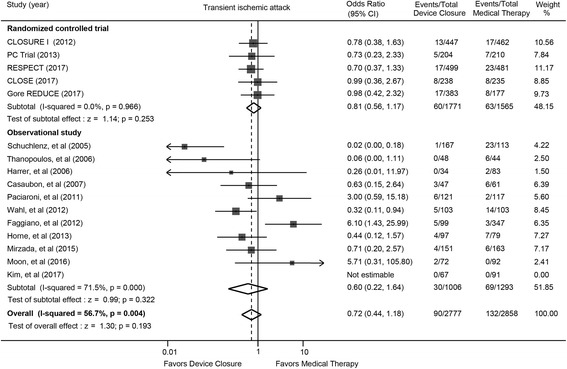
Transient ischemic attack with device closure versus medical therapy. Gore CLOSURE I = Evaluation of the STARFlex Septal Closure System in Patients with a Stroke and/or Transient Ischemic Attack due to Presumed Paradoxical Embolism; CLOSE = the Patent Foramen Ovale Closure or Anticoagulants versus Antiplatelet Therapy to Prevent Stroke Recurrence; PC = Percutaneous Closure of Patent Foramen Ovale in Cryptogenic Embolism; REDUCE = Gore HELEX Septal Occluder and Antiplatelet Medical Management for Reduction of Recurrent Stroke or Imaging-Confirmed TIA in Patients with Patent Foramen Ovale; RESPECT = Randomized Evaluation of Recurrent Stroke Comparing PFO Closure to Established Current Standard of Care Treatment

The overall incidences of TIA was not statistically different between device closure and medical therapy (OR: 0.72; 95% CI: 0.44 to 1.18; *P* = 0.193) (Fig. 3). The lack of statistically significant difference was consistent in randomized trials (OR: 0.81; 95% CI: 0.56 to 1.17; *P* = 0.253) and observational studies (OR: 0.60; 95% CI: 0.22 to 1.64; *P* = 0.322).

### All-cause death

**Fig. 4:**
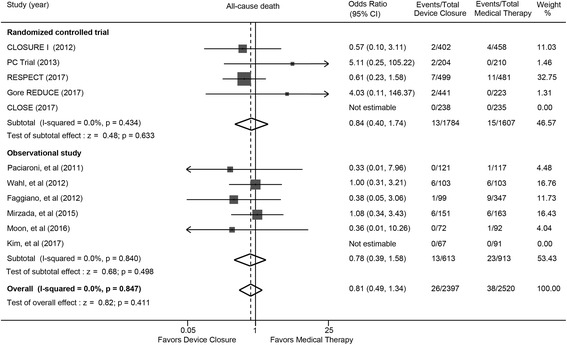
All-cause death with device closure versus medical therapy. Gore CLOSURE I = Evaluation of the STARFlex Septal Closure System in Patients with a Stroke and/or Transient Ischemic Attack due to Presumed Paradoxical Embolism; CLOSE = the Patent Foramen Ovale Closure or Anticoagulants versus Antiplatelet Therapy to Prevent Stroke Recurrence; PC = Percutaneous Closure of Patent Foramen Ovale in Cryptogenic Embolism; REDUCE = Gore HELEX Septal Occluder and Antiplatelet Medical Management for Reduction of Recurrent Stroke or Imaging-Confirmed TIA in Patients with Patent Foramen Ovale; RESPECT = Randomized Evaluation of Recurrent Stroke Comparing PFO Closure to Established Current Standard of Care Treatment

Pooled all-cause mortality was similar between device closure and medical therapy (OR: 0.81; 95% CI: 0.49 to 1.34; *P* = 0.411) (Fig. 4). Consistent findings were found in randomized trials (OR: 0.84; 95% CI: 0.40 to 1.74; *P* = 0.633) and observational studies (OR: 0.78; 95% CI: 0.49 to 1.34; *P* = 0.847).

### Atrial fibrillation or atrial flutter, and pulmonary embolism

**Fig. 5:**
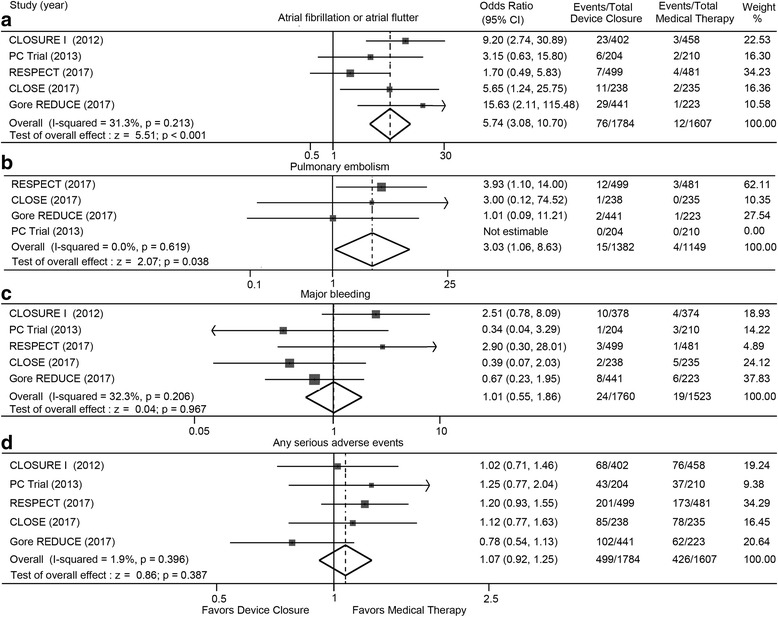
Adverse events with device closure versus medical therapy. Atrial fibrillation or atrial flutter (**a**), pulmonary embolism (**b**), major bleeding (**c**) and any serious adverse events (**d**) with device closure versus medical therapy. Gore CLOSURE I = Evaluation of the STARFlex Septal Closure System in Patients with a Stroke and/or Transient Ischemic Attack due to Presumed Paradoxical Embolism; CLOSE = the Patent Foramen Ovale Closure or Anticoagulants versus Antiplatelet Therapy to Prevent Stroke Recurrence; PC = Percutaneous Closure of Patent Foramen Ovale in Cryptogenic Embolism; REDUCE = Gore HELEX Septal Occluder and Antiplatelet Medical Management for Reduction of Recurrent Stroke or Imaging-Confirmed TIA in Patients with Patent Foramen Ovale; RESPECT = Randomized Evaluation of Recurrent Stroke Comparing PFO Closure to Established Current Standard of Care Treatment

Pooled analysis from randomized trials showed that percutaneous closure significantly increased the risk for atrial fibrillation or atrial flutter (OR: 5.74; 95% CI: 3.08 to 10.70; *P* < 0.001), and pulmonary embolism (OR: 3.03; 95% CI: 1.06 to 8.63; *P* = 0.038) compared with medical therapy (Fig. 5a and b). The number needed to harm was 30 and 143 respectively.

### Major bleeding, and any serious adverse events

The incidences of major bleeding (OR: 1.01; 95% CI: 0.55 to 1.86; *P* = 0.967) and any serious adverse events (OR: 1.07; 95% CI: 0.92 to 1.25; *P* = 0.387) were similar between device closure and medical therapy (Fig. 5c and d).

### Grading of evidence

Based on GRADE summaries (Table 2), we deemed the quality of the evidence to be high for atrial fibrillation or atrial flutter, low for major bleeding, and moderate for other major outcomes. Reasons for rating down were provided in Table 2.

**Table 2:** GRADE assessment of confidence in estimates of effect in randomized trials

Outcome	No. of participants (trials)	Risk of bias	Consistency	Directness	Precision	Publication bias	Quality	Odds ratio (95% CI)
Composite outcome	2776 (4)	No serious limitations	No serious limitations	Serious limitations^§^	No serious limitations	Not detected	Moderate	0.62 (0.44, 0.88)
Recurrent ischemic stroke	3440 (5)	No serious limitations	Serious limitations^†^	No serious limitations	No serious limitations	Not detected	Moderate	0.41 (0.19, 0.89)
TIA	3336 (5)	No serious limitations	No serious limitations	No serious limitations	Serious limitations^¶^	Not detected	Moderate	0.81 (0.56, 1.17)
All-cause death	3391 (5)	No serious limitations	No serious limitations	No serious limitations	Serious limitations^¶^	Not detected	Moderate	0.84 (0.40, 1.74)
Atrial fibrillation or atrial flutter	3391 (5)	No serious limitations	No serious limitations^‡^	No serious limitations	No serious limitations	Not detected	High	5.74 (3.08, 10.70)
Pulmonary embolism	2531 (4)	No serious limitations	No serious limitations	No serious limitations	Serious limitations^¶^	Not detected	Moderate	3.03 (1.06, 8.63)
Major bleeding	3283 (5)	No serious limitations	No serious limitations^‡^	Serious limitations^§^	Serious limitations^¶^	Not detected	Low	1.01 (0.55, 1.86)

*GRADE* Grading of Recommendations Assessment, Development and Evaluation, *OR* odds ratio, *TIA* transient ischemic attack

^†^Moderate to substantial heterogeneity: I^2^ = 59%

^‡^I^2^ = 31 and 32% respectively. Did not downgrade for mild heterogeneity

^§^Definitions of the composite outcome and major bleeding varied across trials. In 2 trials, peripheral embolism or systemic embolism was included in the definition of composite outcome

^¶^95% confidence interval (CI) suggests potential for benefit and harm. Low number of outcome events

### Major subgroup analysis

We performed separate analysis for major outcomes stratified by study designs, number of patients and duration of follow-up. All tested summary effects, which included the composite outcomes, recurrent ischemic stroke, TIA and all-cause death, did not differ significantly in these stratified subgroups (Additional file 2: Table S7). We also performed pooled analyses of prespecified subgroup data from randomized trials, and found significantly lower incidence of the composite outcome only in patients with large shunt size, and lower incidence of recurrent stroke in patients with large shunt size and those present with atrial septal aneurysm, but no significant interaction was detected (Additional file 2: Table S8 and S9).

### Additional analyses

There was no evidence of publication bias for all outcome assessment. Meta-regression did not detect significant confounding effect of the aforementioned covariates on all outcomes (Additional file 2: Table S10).

## Discussion

Our analysis with data from 20 studies and 6961 patients, demonstrated that in patients with a PFO who had a cryptogenic ischemic stroke, PFO closure was associated with a significantly lower incidence of the composite outcome of ischemic stroke, TIA, or all-cause death as compared with medical therapy (moderate-quality evidence), mainly driven by protection against recurrent ischemic stroke. PFO closure increased the risks for atrial fibrillation or atrial flutter (high-quality evidence) and pulmonary embolism (moderate-quality evidence). The risks for TIA, all-cause death, major bleeding and any serious adverse events were not different. Analyses limited to RCTs closely mirrored these results, as did a series of subgroup analyses and meta-regression analyses.

Several meta-analyses on this topic have been published, but all were pooled from 3 trials published in 2012 and 2013 [8–11]. These meta-analyses, with small-to-moderate sample size (~ 2300 patient in total), were inadequately powered to draw conclusions on rare individual outcomes such as stroke. We included a number of recently published high-quality RCTs and observational studies, thus had a much larger sample size and an enhanced statistical power. A series of subgroup analyses with similar results supported the robustness of our findings. We also graded the quality of evidence using appropriate methodology (GRADE).

We showed that PFO closure might increase the likelihood of AF or atrial flutter (most was periprocedure), but the real burden of AF cannot be determined from our analysis because these included trials did not use continuous monitoring to monitor subclinical AF episodes. Subclinical episodes of AF were not uncommon and were associated with a significantly increased risk of stroke [38]. Also data about the autonomic function of the heart and heart rate variability was not reported, it would be interesting to have these data as autonomic dysfunction significantly contributed to silent AF [39]. Nevertheless, atrial fibrillation in our analysis seems not to increase the overall risk of stroke, as the overall risk for stroke was actually significantly reduced.

In the RESPECT trial, the benefit of PFO closure as compared with medical therapy was greater among patients receiving antiplatelet than anticoagulant therapy in the medical-therapy group [7, 20]. A meta-analysis with 8 studies also showed that anticoagulant therapy with warfarin might be superior to antiplatelet therapy in preventing recurrent stroke or TIA in patients with PFO [40]. However, there is a body of evidence not corroborating these findings. A comprehensive meta-analysis with individual participant data from 12 databases did not report a difference in composite outcome of recurrent stroke, TIA or death, or the individual outcome of stroke alone [41]. The very recently released interim analysis of the NAVIGATE ESUS trial, which compared novel oral anticoagulants rivaroxaban with aspirin in patients with cryptogenic embolic stroke, did not detect a difference in rate of stroke or systemic embolism after enrolling 7214 patients [42]. Also in our meta-regression analysis, no interaction was detected between these 2 medical treatment options with respect to the primary and secondary outcomes. As such, currently available data do not provide definite conclusions on whether antiplatelet or anticoagulation medications are superior for patients with a PFO and stroke [43].

In the setting of PFO and concurrent deep venous thrombosis (DVT) without cancer, in which patients are indicated to receive anticoagulant therapy (dabigatran, rivaroxaban, apixaban, or edoxaban over vitamin K antagonist), the most recent 2014 American Heart Association (AHA) and American Stroke Association guideline recommended that PFO closure might be considered, depending on the risk of recurrent DVT [44]. It should be noted that our observations were obtained on the basis that the vast majority of patients (~ 98%) did not have DVT, in which most patients received antiplatelet therapy in the medical therapy. The American guidelines, however, did not recommend PFO closure in these set of patients (class III, level of evidence A). It is important to realize that the guideline was written when the current evidence was yet not available. Based on the updated evidence from randomized trials and our meta-analysis, PFO closure might be an alternative in young patients with PFO and cryptogenic stroke without DVT, particular in but not limited to those concomitant with the presence of an atrial septal aneurysm or large shunt size. Whether benefits of closure would be achieved in other subgroup populations awaits further study. It is important to take into account the benefits in reducing risks for stroke but also the harm in increasing pulmonary embolism.

There are several limitations in our study. First, most observational studies included in our meta-analysis were not adjusted for confounding factors. Second, definitions of the outcomes of interest were not identical across studies. Third, substantial heterogeneity was observed in several analyses. Fourth, results of meta-regression analyses and prespecified subgroup data can only be considered exploratory. Fifth, performance of different device cannot be performed. Finally, our findings cannot be generalized to patients older than 60 years of age.

## Conclusions

Compared with medical therapy for the secondary prevention of cryptogenic stroke, our study showed moderate-quality evidence that PFO closure was associated with significantly lower incidence of the composite outcome of ischemic stroke, TIA, or all-cause death, which was mainly driven by lower risk for ischemic stroke. However, PFO closure increased the risks for atrial fibrillation or atrial flutter (high-quality evidence) and pulmonary embolism (moderate-quality evidence). The risks for TIA, all-cause death, and major bleeding were similar.

## Additional files

Additional file 1: PRISMA 2009 Checklist. (DOCX 29 kb)

Additional file 2: **Figure S1.** Flow diagram of study selection. **Table S1.** Main inclusion and exclusion criteria of included randomized trials. **Table S2.** Definitions of degree of shunting and atrial septal aneurysm in randomized trials. **Table S3.** Primary and secondary endpoints of included randomized trials. **Table S4.** Definitions of composite outcome, major bleeding, recurrent stroke and TIA in randomized trials. **Table S5.** Risk of bias of included randomized trials. **Table S6.** Study quality of included comparative observational studies using the Newcastle-Ottawa scale. **Figure S2.** The composite outcome of recurrent stroke, TIA and all-cause death with device closure versus medical therapy from randomized controlled trials and adjusted observational studies. **Table S7.** Subgroup analysis of the major outcomes based on study designs, number of patients and duration of follow-up. **Table S8.** Subgroup analysis of the composite outcome in randomized trials. **Table S9.** Subgroup analysis of recurrent ischemic stroke in randomized trials. **Table S10.** Meta-regression analysis in randomized trials exploring the potential for effect modification by multiple variables, including Moderate to severe PFO, atrial septal aneurysm, index event of stroke, and anticoagulation in medical treatment. (DOCX 594 kb)

## Abbreviations

CI: confidence interval
GRADE: Grading of Recommendations, Assessment, Development and Evaluation
OR: odds ratio
PFO: patent foramen ovale
RCT: randomized controlled trial
TIA: transient ischemic attack
