# Supplementary material for: Percutaneous closure versus medical therapy for stroke with patent foramen Ovale: a systematic review and meta-analysis
Source: BMC Cardiovasc Disord. 2018 Mar 2;18:45. doi: 10.1186/s12872-018-0780-x (PMC5834900; doi:10.1186/s12872-018-0780-x)
Supplement: Supplementary file 2 — Figure S1. Flow diagram of study selection. Table S1. Main inclusion and exclusion criteria of included randomized trials. Table S2. Definitions of degree of shunting and atrial septal aneurysm in randomized trials. Table S3. Primary and secondary endpoints of included randomized trials. Table S4. Definitions of composite outcome, major bleeding, recurrent stroke and TIA in randomized trials. Table S5. Risk of bias of included randomized trials. Table S6. Study quality of included comparative observational studies using the Newcastle-Ottawa scale. Figure S2. The composite outcome of recurrent stroke, TIA and all-cause death with device closure versus medical therapy from randomized controlled trials and adjusted observational studies. Table S7. Subgroup analysis of the major outcomes based on study designs, number of patients and duration of follow-up. Table S8. Subgroup analysis of the composite outcome in randomized trials. Table S9. Subgroup analysis of recurrent ischemic stroke in randomized trials. Table S10. Meta-regression analysis in randomized trials exploring the potential for effect modification by multiple variables, including Moderate to severe PFO, atrial septal aneurysm, index event of stroke, and anticoagulation in medical treatment. (DOCX 594 kb) [file 12872_2018_780_MOESM2_ESM.docx]

**Supplementary Appendix**

**Percutaneous Closure versus Medical Therapy for Stroke with Patent Foramen Ovale: Systematic Review and Meta-analysis**

Brief title: PFO Closure vs. Medical Therapy After Stroke


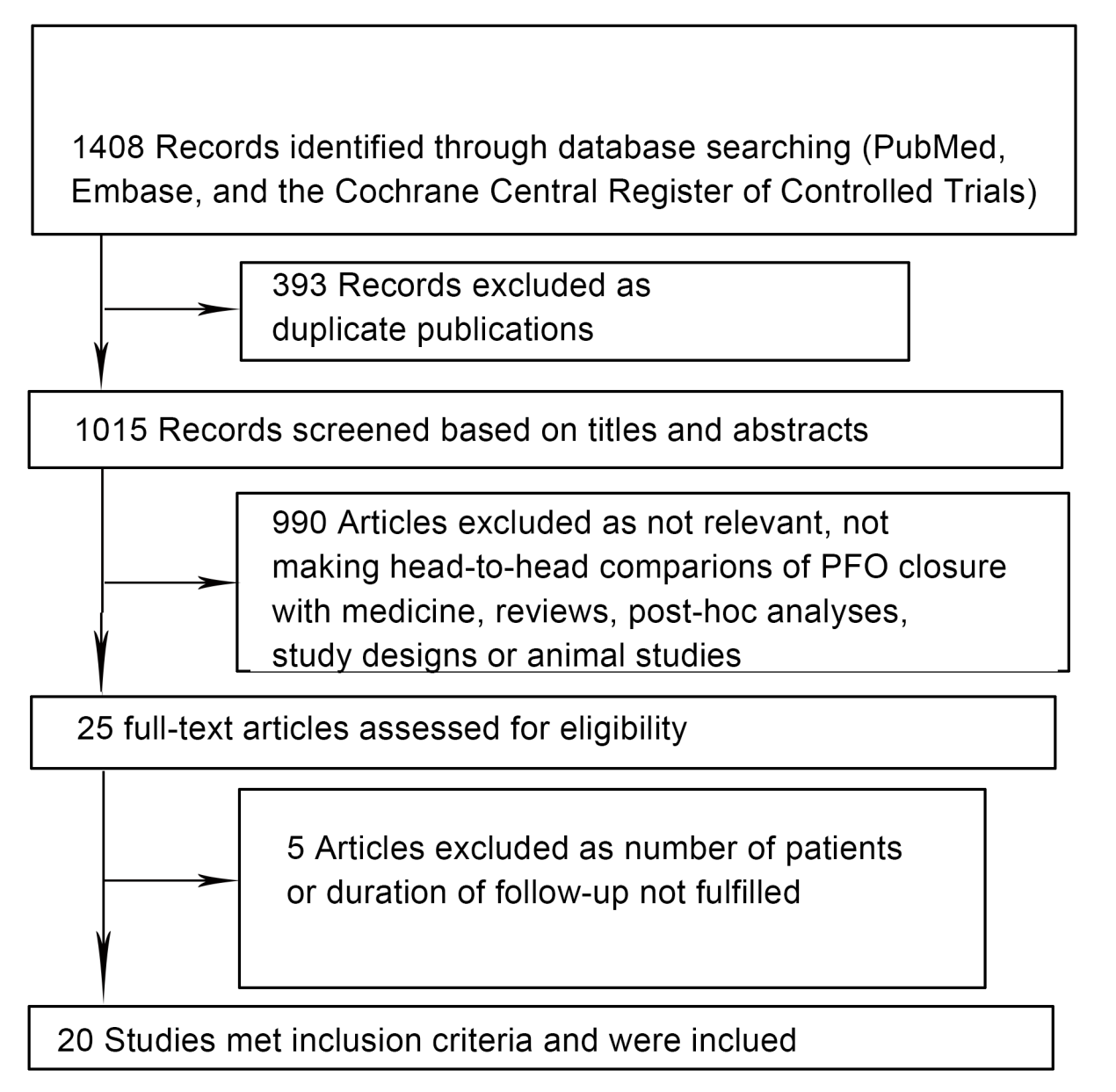


**Figure S1.** Flow diagram of study selection.

**Table S1.** Main inclusion and exclusion criteria of included randomized trials.

| Trial | Year | Main inclusion criteria | Main exclusion criteria |
| --- | --- | --- | --- |
| CLOSURE I | 2012 | Patients 18 and 60 years of age, had had an ischemic stroke or TIA within the previous 6 months, and had evidence of a patent foramen ovale, as documented by transesophageal echocardiography with a bubble study (i.e., with the injection of agitated saline) showing right-to-left shunting at the atrial level during a Valsalva maneuver. | Any identified potential cause of ischemic stroke or TIA other than the patent foramen ovale, such as clinically significant carotid-artery stenosis, complex aortic-arch atheroma, clinically significant left ventricular dysfunction or left ventricular aneurysm, or atrial fibrillation. |
| PC Trial | 2013 | Patients less than 60 years of age with a patent foramen ovale documented on transesophageal echocardiography with a right-to-left shunt during the bubble test or color Doppler flow imaging either spontaneously or with a Valsalva or cough maneuver, and no other identifiable cause of stroke or peripheral thromboembolism were eligible for the study if they presented with clinically and neuroradiologically verified ischemic stroke, a TIA with a neuroradiologically verified cerebral ischemic lesion, or a clinically and radiologically verified extracranial peripheral thromboembolic event. | Any identifiable cause for the thromboembolic event other than PFO, such as mural thrombus, dilated cardiomyopathy, prosthetic heart valve, mitral stenosis, bacterial and nonbacterial endocarditis, cardiac myxoma, atherosclerosis of the aorta, chronic or paroxysmal atrial fibrillation, significant atherosclerosis or dissection of the aorta, clinically relevant atherosclerosis or dissection of the intra- and extracranial arteries, hyperviscosity syndromes. |
| RESPECT | 2017 | Patients 18 and 60 years of age, had had a cryptogenic ischemic stroke, and had a patent foramen ovale identified by means of transesophageal echocardiography. | A mechanism for the index stroke other than paradoxical embolization could be identified, such as large-vessel disease, any cardioembolic source, a lacunar infarct that was probably due to intrinsic small-vessel disease, or an arterial hypercoagulable state (as indicated by the presence of anticardiolipin antibody, lupus anticoagulant, or hyperhomocysteinemia) |
| CLOSE | 2017 | Patients 16 to 60 years of age were eligible for the trial if they had had an ischemic stroke within the previous 6 months with no identifiable cause other than a PFO with an associated atrial septal aneurysm or large interatrial shunt. | Another cause for stroke associated with PFO, Isolated ASD or ASD associated with PFO but with a hemodynamically significant left-to-right shunt requiring closure. |
| Gore REDUCE | 2017 | Patients 18 to 59 years of age, had had a cryptogenic ischemic stroke within 180 days before randomization, and had a PFO with a right-to-left shunt. | Patient has other potential source(s) of cardio-embolism, for example: atrial fibrillation (AFib) or atrial flutter (AFlu), prosthetic heart valve, severe native valve disease, left ventricular ejection fraction of <40%, severe ventricular wall motion abnormalities (akinesis, severe hypokinesis), intracardiac thrombus, mitral valve stenosis, prior cardiac surgery, other major congenital cardiac abnormality. |

**Table S2.** Definitions of degree of shunting and atrial septal aneurysm in randomized trials.

| Trial | Year | Degree of Shunting | Atrial septal aneurysm |
| --- | --- | --- | --- |
| CLOSURE I | 2012 | None, trace (1-10 bubbles), moderate (10-25 bubbles), and substantial (>25 bubbles). | A hypermobile septum primum associated with a PFO, as demonstrated by total septal mobility of 10mm or greater. |
| PC Trial | 2013 | Grade 0=none, grade 1=minimal (1-5 bubbles), grade 2=moderate (6-20 bubbles), and grade 3=severe (>20 bubbles). | A protrusion of the interatrial septum, or part of it, of more or equal than 15mm beyond the plane of the interatrial septum and the diameter of the aneurysm base measured at least 15mm. |
| RESPECT | 2017 | Grade 1 indicated 1 to 9 bubbles; grade 2, 10 to 20 bubbles; and grade 3, more than 20 bubbles. | A septum primum excursion of 10 mm or more. |
| CLOSE | 2017 | Large shunt was defined by the appearance of more than 30 microbubbles in the left atrium within three cardiac cycles after opacification of the right atrium. | A septum primum excursion greater than 10 mm. |
| Gore REDUCE | 2017 | Grade 0=none, small (1-5 bubbles), moderate (6-25 bubbles), and large (>25 bubbles). | NA |

**Table S3.** Primary and secondary endpoints of included randomized trials.

| Trial | Year | Primary endpoint | Secondary endpoints |
| --- | --- | --- | --- |
| CLOSURE I | 2012 | A composite of stroke or TIA during 2 years of follow-up, death from any cause during the first 30 days, and death from neurologic causes between 31 days and 2 years. | Major bleeding, death from any cause, stroke, TIA, and transient neurologic events of uncertain cause |
| PC Trial | 2013 | A composite of death, nonfatal stroke, TIA, or peripheral embolism. | Death from any cause, stroke, TIA, cardiovascular death, atrial fibrillation, myocardial infarction, hospitalization related to the patent foramen ovale or its treatment, device problems, and bleeding |
| RESPECT | 2017 | A composite of recurrent nonfatal ischemic stroke, fatal is chemic stroke, or early death after randomization | complete closure of the patent foramen ovale on the 6-month follow-up transesophageal echocardiogram, the absence of recurrent symptomatic nonfatal ischemic stroke or cardiovascular death, and the absence of a transient ischemic attack |
| CLOSE | 2017 | Fatal or nonfatal stroke | The composite of ischemic stroke, transient ischemic attack, or systemic embolism; disabling stroke; ischemic stroke; cerebral hemorrhage; transient ischemic attack; systemic embolism; all-cause mortality; death from vascular-related causes; success of device implantation; and success of PFO closure. |
| Gore REDUCE | 2017 | Ischemic stroke, a composite of clinical ischemic stroke or silent brain infarction | Deaths and suspected recurrent stroke or transient ischemic attack |

**Table S4.** Definitions of composite outcome, major bleeding, recurrent stroke and TIA in randomized trials.

| Trial | Year | Composite outcome | Major bleeding | Stroke | TIA |
| --- | --- | --- | --- | --- | --- |
| CLOSURE I | 2012 | A composite of stroke or TIA during 2 years of follow-up, death from any cause during the first 30 days, and death from neurologic causes between 31 days and 2 years. | The occurrence of intracranial, intraocular, or retroperitoneal hemorrhage or any hemorrhage requiring a transfusion or resulting in a hematocrit decrease of > 15% or hemoglobin decrease of > 5 g/dL | An acute focal neurological event that is MR imaging positive, regardless of duration of clinical symptoms. | A sudden focal neurological event lasting at least 10 minutes without evidence of acute ischemic brain injury on DWMR imaging and consisting of hemiplegia/paresis, onoplegia/paresis, quadriplegia/paresis, language disturbance other than isolated slurred speech, blindness in one or both eyes, or significant difficulty walking. |
| PC Trial | 2013 | A composite of death, nonfatal stroke, TIA, or peripheral embolism | Bleeding requiring any blood transfusion | A stroke deemed to have caused death either directly by brain damage or indirectly by some non-neurological complication, any neurologic deficit lasting for >24 hours typically with documentation in magnet resonance imaging (MRI) or computer tomography (CT). | A temporary neurologic deficit presumably due to reduced blood flow in a particular cerebral artery lasting for ≤24 hours with complete resolution of the neurologic deficit. |
| RESPECT | 2017 | A composite of recurrent nonfatal ischemic stroke, fatal ischemic stroke, or early death | NA | NA | NA |
| CLOSE | 2017 | A composite of death, ischemic stroke, transient ischemic attack, or systemic embolism | Transfusion ≤3 units of packed cells (or equivalent of whole blood), or requiring hospitalization (or prolonging hospitalization), or requiring surgical treatment, etc. | Acute focal neurologic symptoms with a recent cerebral infarct, as identified on brain imaging, regardless of the duration of the symptoms. | Sudden onset of neurological symptoms, presumed to be ischemic, resolving in less than 24 hours, clearly attributable to focal involvement of the central nervous system (or of the eye) with no signs of a corresponding recent cerebral infarction on brain imaging. |
| Gore REDUCE | 2017 | NA | NA | An acute focal neurologic deficit, presumably due to ischemia, that either resulted in clinical symptoms lasting 24 hours or more or was associated with evidence of relevant infarction on magnetic resonance imaging (MRI) or — if MRI could not be performed — computed tomography (CT) of the brain. | NA |

**Table S5.** Risk of bias of included randomized trials.

| Trial | Random sequence generation (selection bias) | Allocation concealment (selection bias) | Blinding of participants and personnel (performance bias) | Blinding of outcome assessment (detection bias) | Incomplete outcome data (attrition bias) | Selective reporting (reporting bias) | Other bias |
| --- | --- | --- | --- | --- | --- | --- | --- |
| CLOSURE I | Low risk | Low risk | High risk | Low risk | Low risk | Low risk | Low risk |
| PC Trial | Low risk | Low risk | High risk | Low risk | Low risk | Low risk | Low risk |
| RESPECT | Unclear risk | Unclear risk | High risk | Low risk | Unclear risk | Low risk | Low risk |
| CLOSE | Low risk | Low risk | High risk | Low risk | Low risk | Low risk | Low risk |
| Gore REDUCE | Unclear risk | Unclear risk | High risk | Low risk | Low risk | Low risk | Low risk |

**Table S6.** Study quality of included comparative observational studies using the Newcastle-Ottawa scale.

| **Author/Study** | **Year** | **Study quality (Newcastle-Ottawa Scale)** | | | |
| --- | --- | --- | --- | --- | --- |
|  |  | Selection | Comparability | Outcome/exposure | Total score |
| Wahl, et al | 2012 | ******** | ****** | ******* | 9 |
| Alushi, et al | 2014 | ******** | ***** | ******* | 8 |
| Pezzini, et al | 2016 | ******** | ***** | ******* | 8 |
| Casaubon, et al | 2007 | ******** |  | ******* | 7 |
| Faggiano, et al | 2012 | ******** |  | ******* | 7 |
| Horner, et al | 2013 | ******** |  | ******* | 7 |
| Kim, et al | 2017 | ******** |  | ****** | 6 |
| Mazzucco, et al | 2012 | ******** |  | ******* | 7 |
| Lee, et al | 2010 | ******** |  | ******* | 7 |
| Moon, et al | 2016 | ******** |  | ****** | 6 |
| Mirzada, et al | 2015 | ******** |  | ******* | 7 |
| Schuchlenz, et al | 2005 | ******** |  | ******* | 7 |
| Paciaroni, et al | 2011 | ******** |  | ******* | 7 |
| Thanopoulos, et al | 2006 | ******** |  | ******* | 7 |
| Harrer, et al | 2006 | ******** |  | ******* | 7 |


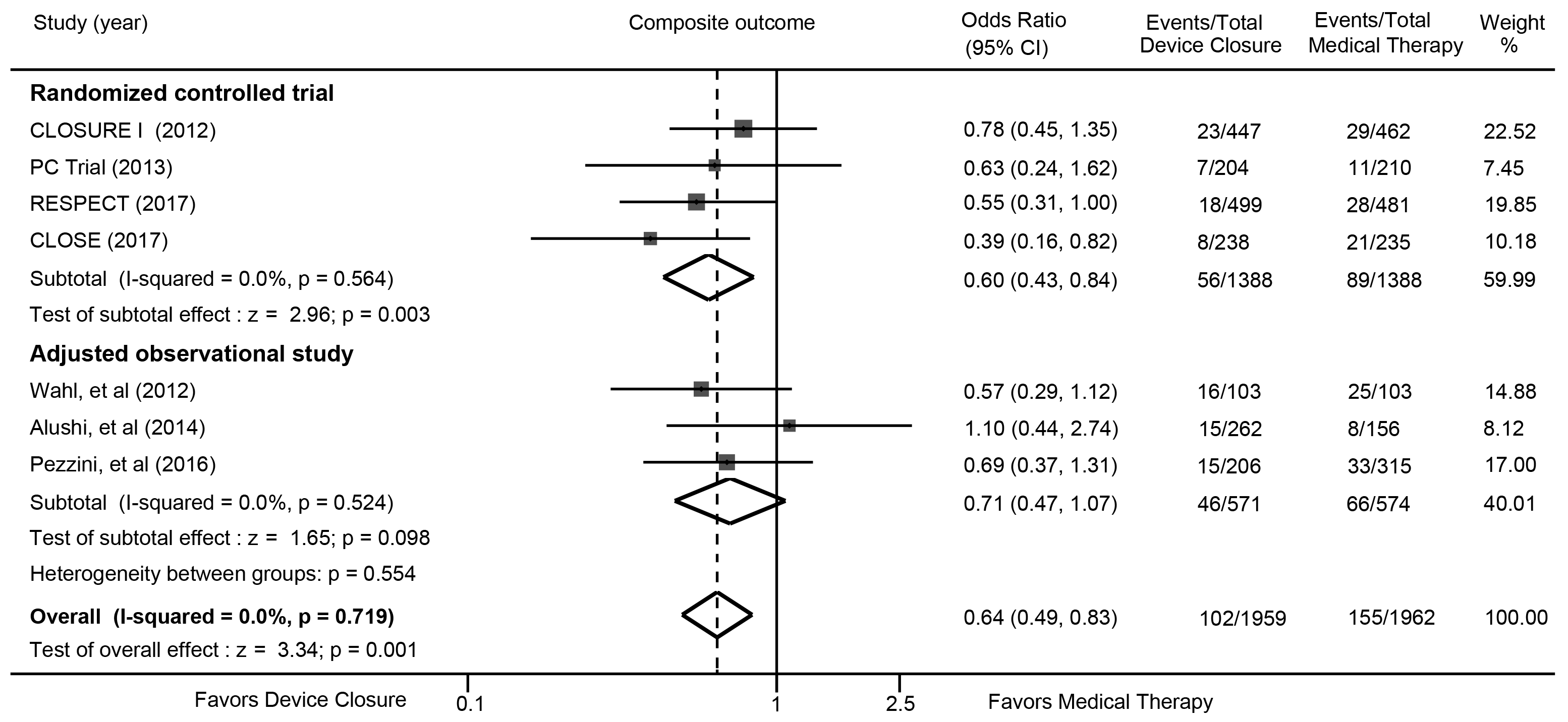


**Figure S2**. The composite outcome of recurrent stroke, TIA and all-cause death with device closure versus medical therapy from randomized controlled trials and adjusted observational studies.

**Table S7.** Subgroup analysis of the major outcomes based on study designs, number of patients and duration of follow-up.

| **Composite outcome** |  | No. of trials | Events/Total (Closure) | Events/Total (Medical) | OR (95% CI) | P value | I2 | P value of heterogeneity | P value for interaction |
| --- | --- | --- | --- | --- | --- | --- | --- | --- | --- |
|  | Overall | 16 | 139/2765 | 263/3073 | 0.57 (0.38, 0.85) | 0.01 | 61.9 | 0.001 |  |
| Study design | RCT | 4 | 56/1388 | 89/1388 | 0.62 (0.44, 0.88) | 0.01 | 0.0 | 0.46 | 0.90 |
|  | Observational study | 12 | 83/1377 | 174/1685 | 0.53 (0.29, 0.97) | 0.04 | 70.7 | <0.001 |  |
| Number of patients | < 400 | 9 | 47/810 | 127/867 | 0.35 (0.17, 0.74) | 0.01 | 66.9 | 0.002 | 0.14 |
|  | ≥ 400 | 7 | 92/1955 | 136/2206 | 0.77 (0.51, 1.16) | 0.22 | 49.7 | 0.06 |  |
| Follow-up | < 3y | 7 | 38/969 | 104/980 | 0.27 (0.09, 0.78) | 0.02 | 77.1 | <0.001 | 0.12 |
|  | ≥ 3y | 9 | 101/1796 | 159/2093 | 0.72 (0.51, 1.01) | 0.06 | 34.2 | 0.14 |  |
| **Recurrent ischemic stroke** |  |  |  |  |  |  |  |  |  |
|  | Overall | 18 | 55/2907 | 149/3115 | 0.39 (0.24, 0.63) | <0.001 | 33.4 | 0.08 |  |
| Study design | RCT | 5 | 37/1829 | 72/1611 | 0.41 (0.19, 0.89) | 0.03 | 58.9 | 0.05 | 0.74 |
|  | Observational study | 13 | 18/1078 | 77/1504 | 0.36 (0.19, 0.70) | 0.002 | 21.3 | 0.23 |  |
| Number of patients | < 400 | 12 | 17/979 | 77/1157 | 0.35 (0.20, 0.64) | 0.001 | 7.5 | 0.37 | 0.39 |
|  | ≥ 400 | 6 | 38/1928 | 72/1958 | 0.47 (0.21, 1.06) | 0.07 | 59.4 | 0.03 |  |
| Follow-up | < 3y | 9 | 15/1116 | 53/1111 | 0.22 (0.09, 0.56) | 0.002 | 38.2 | 0.11 | 0.23 |
|  | ≥ 3y | 9 | 40/1791 | 96/2004 | 0.50 (0.28, 0.88) | 0.16 | 32.6 | 0.16 |  |
| **Transient ischemic attack** |  |  |  |  |  |  |  |  |  |
|  | Overall | 16 | 90/2777 | 132/2858 | 0.72 (0.44, 1.18) | 0.19 | 56.7 | 0.004 |  |
| Study design | RCT | 5 | 60/1771 | 63/1565 | 0.81 (0.56, 1.17) | 0.25 | 0 | 0.97 | 0.67 |
|  | Observational study | 11 | 30/1006 | 69/1293 | 0.60 (0.22, 1.64) | 0.32 | 71.5 | <0.001 |  |
| Number of patients | < 400 | 6 | 25/907 | 66/946 | 0.45 (0.18, 1.13) | 0.09 | 60.6 | 0.009 | 0.18 |
|  | ≥ 400 | 9 | 65/1870 | 66/1912 | 0.97 (0.61, 1.53) | 0.88 | 34.2 | 0.18 |  |
| Follow-up | < 3y | 8 | 29/1066 | 61/1059 | 0.51 (0.17, 1.54) | 0.23 | 71.1 | 0.002 | 0.47 |
|  | ≥ 3y | 8 | 61/1711 | 71/1739 | 0.84 (0.51, 1.38) | 0.5 | 37.9 | 0.13 |  |
| **All-cause death** |  |  |  |  |  |  |  |  |  |
|  | Overall | 11 | 26/2397 | 38/2520 | 0.81 (0.49, 1.34) | 0.41 | 0 | 0.85 |  |
| Study design | RCT | 5 | 13/1784 | 15/1607 | 0.84 (0.40, 1.74) | 0.63 | 0 | 0.43 | 0.88 |
|  | Observational study | 6 | 13/613 | 23/913 | 0.78 (0.39, 1.58) | 0.5 | 0 | 0.84 |  |
| Number of patients | < 400 | 5 | 12/514 | 14/566 | 0.90 (0.42, 1.92) | 0.78 | 0 | 0.85 | 0.62 |
|  | ≥ 400 | 6 | 14/1883 | 24/1954 | 0.75 (0.38, 1.47) | 0.4 | 0 | 0.5 |  |
| Follow-up | < 3y | 4 | 2/662 | 6/758 | 0.47 (0.12, 1.83) | 0.28 | 0 | 0.94 | 0.45 |
|  | ≥ 3y | 7 | 24/1735 | 32/1762 | 0.89 (0.52, 1.54) | 0.41 | 0 | 0.65 |  |

**Table S8.** Subgroup analysis of the composite outcome in randomized trials.

| Subgroup |  | No. of trials | Events/Total (Closure) | Events/Total (Medical) | OR (95% CI) | P value | I2 | P value of heterogeneity | P value for interaction |
| --- | --- | --- | --- | --- | --- | --- | --- | --- | --- |
| Age | < 45y | 2 | 5/321 | 11/307 | 0.43 (0.15, 1.26) | 0.13 | 24.9 | 0.25 | 0.65 |
|  | ≥ 45y | 2 | 11/375 | 16/379 | 0.68 (0.31, 1.50) | 0.34 | 30.0 | 0.23 |  |
| Gender | Male | 2 | 12/476 | 25/500 | 0.50 (0.25, 1.00) | 0.05 | 0 | 0.97 | 0.14 |
|  | Female | 2 | 19/423 | 20/432 | 1.03 (0.54, 1.96) | 0.93 | 0 | 0.35 |  |
| Atrial septal aneurysm | Present | 2 | 11/198 | 11/211 | 1.07 (0.45, 2.52) | 0.88 | 0 | 0.32 | 0.51 |
|  | Absent | 2 | 18/406 | 29/450 | 0.69 (0.38, 1.25) | 0.22 | 40.1 | 0.20 |  |
| Entry event | Stroke | 2 | 20/465 | 23/487 | 0.91 (0.49, 1.68) | 0.76 | 0 | 0.40 | 0.57 |
|  | TIA | 2 | 9/139 | 17/173 | 0.64 (0.27, 1.48) | 0.29 | 0 | 0.80 |  |
| Shunt size | Large | 2 | 5/334 | 13/296 | 0.32 (0.11, 0.91) | 0.03 | 35.5 | 0.21 | 0.14 |
|  | Small to moderate | 2 | 22/509 | 28/562 | 0.90 (0.51, 1.60) | 0.71 | 0 | 0.60 |  |

**Table S9.** Subgroup analysis of recurrent ischemic stroke in randomized trials.

| Subgroup |  | No. of trials | Events/Total (Closure) | Events/Total (Medical) | OR (95% CI) | P value | I2 | P value of heterogeneity | P value for interaction |
| --- | --- | --- | --- | --- | --- | --- | --- | --- | --- |
| Age | < 45y | 3 | 9/558 | 21/436 | 0.37 (0.17, 0.82) | 0.02 | 0.0 | 0.41 | 0.78 |
|  | ≥ 45y | 3 | 15/613 | 33/498 | 0.31 (0.09, 1.09) | 0.07 | 54.5 | 0.11 |  |
| Gender | Male | 3 | 13/666 | 37/548 | 0.24 (0.06, 1.00) | 0.05 | 64.2 | 0.06 | 0.61 |
|  | Female | 3 | 11/512 | 17/391 | 0.50 (0.24, 1.08) | 0.08 | 0 | 0.79 |  |
| Atrial septal aneurysm | Present | 2 | 3/260 | 22/244 | 0.16 (0.05, 0.51) | 0.002 | 1.8 | 0.31 | 0.37 |
|  | Absent | 2 | 15/477 | 20/472 | 0.44 (0.05, 4.27) | 0.48 | 61.1 | 0.11 |  |
| Shunt size | Large | 3 | 9/752 | 31/565 | 0.22 (0.10, 0.47) | <0.001 | 0 | 0.73 | 0.08 |
|  | Small to moderate | 2 | 14/324 | 14/287 | 0.88 (0.34, 2.27) | 0.80 | 10.7 | 0.29 |  |

**Table S10.** Meta-regression analysis in randomized trials exploring the potential for effect modification by multiple variables, including Moderate to severe PFO, atrial septal aneurysm, index event of stroke, and anticoagulation in medical treatment

|  | Moderate to severe PFO | Atrial septal aneurysm | Index event of stroke | Anticoagulation in medical treatment |
| --- | --- | --- | --- | --- |
| Composite outcome | 0.190 | 0.348 | 0.920 | 0.254 |
| Recurrent ischemic stroke | 0.183 | 0.241 | 0.551 | 0.098 |
| Transient ischemic attack | 0.350 | 0.294 | 0.620 | 0.563 |
| All-cause death | 0.458 | 0.743 | 0.956 | 0.587 |
| Atrial fibrillation or atrial flutter | 0.899 | 0.658 | 0.629 | 0.609 |
| Major bleeding | 0.161 | 0.262 | 0.290 | 0.176 |

P values for interaction were provided.
